# Docetaxel versus docetaxel plus cisplatin for non-small-cell lung cancer: a meta-analysis of randomized clinical trials

**DOI:** 10.18632/oncotarget.17071

**Published:** 2017-04-13

**Authors:** Ang Li, Zhi-Jian Wei, Han Ding, Hao-Shuai Tang, Heng-Xing Zhou, Xue Yao, Shi-Qing Feng

**Affiliations:** ^1^ Department of Orthopedics, Tianjin Medical University General Hospital, Heping District, Tianjin, China

**Keywords:** docetaxel, cisplatin, meta-analysis, non-small-cell lung cancer, response rate

## Abstract

**Objective:**

To compare the activity, efficacy and toxicity of docetaxel versus docetaxel plus cisplatin in patients with non-small-cell lung cancer.

**Methods:**

A literature search was performed in the EMBASE, Medline, Cochrane Library, Web of Science, China National Knowledge Internet, Wan-fang databases. The trials that were found were then evaluated for eligibility. The Cochrane Collaboration's Review Manager software was used to perform the meta-analyses.

**Results:**

Nine clinical trials including 1257 patients were included. The docetaxel plus cisplatin regimens had higher overall response rates compared with the docetaxel regimen (RR = 0.70; 95% CI, 0.61 to 0.80; P < 0.00001). No statistically significant difference was observed between the two regimens with respect to the one-year survival rate (RR = 1.04; 95% CI, 0.90 to 1.19; P = 0.62). Patients treated with the DP regimen were more likely to experience anemia, thrombocytopenia, nausea/vomiting, nephrotoxicity, hyponatremia, mucositis and treatment-related deaths compared with patients treated with docetaxel alone. No significant difference was observed between the two regimens with respect to the occurrence of neurotoxicity, diarrhea, fatigue, pneumonitis, neutropenia and leucopenia.

**Conclusions:**

The docetaxel plus cisplatin combination regimen resulted in a high response rate and a high adverse effect rate compared with docetaxel monochemotherapy for non-small-cell lung cancer.

## INTRODUCTION

Non-small-cell lung cancer (NSCLC) is common and accounts for up to 85% of lung cancers [1]. Most patients with NSCLC are diagnosed at an advanced stage, which means that many of these patients lose the opportunity for definitive surgical resection, for which the 5-year survival rate is < 15% [2]. Despite considerable progress in treatment that has been achieved in the last several decades, advanced NSCLC still remains a challenging malignant tumor that is unable to be cured in the majority of patients [3].

Docetaxel (Taxotere), a semi-synthetic taxoid derived from the rare pacific yew tree Taxus Baccata, has demonstrated significant antitumor activity. It is one of the most active single agents in both previously untreated patients and in those who have relapsed or progressed following cisplatin-based chemotherapy [4, 5]. Docetaxel was defined as a new chemotherapy agent according to the American Society of Clinical Oncology (ASCO)[6]. The stabilization of microtubules by docetaxel results in the inhibition of mitotic cell division between metaphase and anaphase, which leads to the initiation of apoptosis. Previous research has shown that single-agent docetaxel at doses of 60, 75 or 100 mg/m^2^ administered once every 3 weeks could lead to objective response rates of approximately 30% in untreated patients with advanced NSCLC [7, 8].

Cisplatin-based doublets are recommended for the adjuvant or neoadjuvant treatment of potentially operable NSCLC and as a first-line therapy for advanced or metastatic NSCLC [9]. Extensive clinical phase II trials in the first-line setting recorded response rates of 32% to 52% and a survival (median, 8 to 12 months) of 33% to 48% [10, 11]. Kubota et al reported that the docetaxel plus cisplatin (DP) regimen was associated with marked improvements in overall survival rates and in quality of life (QOL), compared with the vindesine plus cisplatin regimen. The use of the DP regimen resulted in greater clinical benefits in patients with previously untreated stage IV NSCLC [12]. In addition, the DP regimen was reported to be an effective and well-tolerated regimen in chemo-naive patients with advanced NSCLC [13, 14]. However, for elderly patients or patients with reduced performance status, cisplatin-based protocols are often too toxic and should only be used with caution [15]. Aging is associated with deterioration of renal and liver function, decreased bone marrow reserves and the presence of comorbid illnesses. Moreover, docetaxel monotherapy was reported to be not inferior to DP, with less toxicity and better tolerability in patients with advanced NSCLC [16, 17].

Several RCTs(randomized clinical trials) were performed to evaluate the activity and toxicity of the DP combination as a first-line treatment of chemotherapy-naive patients with metastatic or unresectable locally advanced NSCLC [18–20]. However, the results varied considerably, and the toxic effects of combination therapy such as grade 3-4 neutropenia, myelosuppression, nausea and vomiting were more common after this therapy compared with others. In addition, published studies that have compared platinum-based combinations with the corresponding non-platinum monotherapies demonstrated a higher response rate and higher overall survival rates in the combination arms [21]. The main arguments against the use of chemotherapy in NSCLC are the marginal (if any) improvements in survival and response as well as the occurrence of severe and even unacceptable toxicity profiles.

Accordingly, in this paper, we conducted a meta-analysis to compare the clinical profile of docetaxel monotherapy with that of DP combination chemotherapy in terms of response rate, overall survival and toxicity in patients with NSCLC.

## METHODS

### Literature search

The following electronic databases were independently and extensively searched by two investigators from their inception through May 2016: EMBASE, Medline, the Cochrane Library, Web of Science, the China National Knowledge Internet (CNKI), and the Wan-fang database. The search strategy was based on a combination of three concepts adjusted to each database, when necessary. Concept one included all of the terms for docetaxel, and Concept two included the terms for cisplatin; Concept three included all of the terms for non-small-cell lung cancer. We only accepted one set of data on the same topic. The bibliographies of the included studies and dissertations were also searched for additional publications. All of the eligible studies were identified by two independent authors (AL, ZJW), and any disagreements were settled by consensus or consultation with a third author (HD).

### Study selection

In order to be included in this analysis, the trials had to fulfill the following inclusion criteria: 1) contain patients with histologically proven NSCLC; 2) previous surgery was allowed if it had been completed at least 4 weeks before inclusion; 3) prior radiotherapy, except for that intended for the primary lesion, was permitted if it had been completed at least 2 weeks before inclusion; 3) randomized controlled clinical trials; 4) docetaxel monotherapy and DP doublet regimens were compared as chemotherapy regimens without the addition of confounding agents or interventions; 5) both groups could receive some foundation therapy or supportive care such as granulocyte colony-stimulating factor (G-CSF), antiemetic treatment with ondansetron and dexamethasone. The exclusion criteria were as follows: 1) an evaluation of the activity, efficacy and toxicity of docetaxel versus DP was absent in the studies; 2) agents were used as pre- or post-operative adjuvant chemotherapy; 3) the study featured comparisons of other types of chemotherapy regimens; 4) Early studies published as a series of articles from the same institution or author that contained significant overlapping data were excluded for fear of multiple publication bias; 5) case reports, editorials, experimental studies, conference articles and other studies that failed to provide detailed results were excluded.

### Data extraction

After duplicates were removed and the study selection process was completed, the titles and abstracts were scanned by two independent investigators (AL, ZJW) according to predefined selection criteria, and potentially relevant RCTs were selected. The relevant data were extracted by adopting a predetermined standardized procedure, which involved the first authors, year of publication, country, demographic characteristics of the participants, and the treatment regimen for each group. All data were verified for internal consistency, and controversies were settled by consensus or discussion with a third author. Whenever possible, the first authors were contacted to obtain and clarify the relevant data, when appropriate, as specified by the standardized protocol.

### Quality assessment

The methodological quality of the included trials was assessed by the Cochrane Collaboration's tool [22]. This tool focuses on the internal validity of the trial and on the assessment of the risk of possible bias in different phases of the trial. The following items were assessed: random sequence generation, allocation concealment, blinding of participants, personnel and outcome assessment, incomplete outcome measures, selective outcome reporting and other types of bias. Each item was classified according to the risk of bias; high, low, and unclear risk are represented as High (H), Low (L) and Unclear (U), respectively. All of the eligible studies were identified by two independent authors (AL, ZJW), and any disagreements were settled by consensus or discussion with a third author (HST).

### Outcome assessment

The overall survival (OS) was defined as the time that elapsed from random assignment until death from any cause and was censored at the last follow-up date. Response evaluation was performed according to the Response Evaluation Criteria In Solid Tumors [23]. The overall response rate was defined as the sum of the partial response rate (PR) and the complete response rate (CR). One-year survival rates and overall response rates (CR plus PR) were primary outcomes in the meta-analysis. Symptom scores of quality of life (QOL) were evaluated according to the Functional Assessment of Cancer Therapy-Lung (FACT-L), which consists of a seven-item disease-specific subscale [24]. Toxicity profiles were reported according to the World Health Organization (WHO) criteria or the Cooperative Group Common Toxicity Criteria [25]. According to the National Cancer Institute Common Terminology Criteria (NCI-CTC) for Adverse Events (version 3.0), grade 3 or 4 toxic effects included anemia, leucopenia, neutropenia, febrile neutropenia, thrombocytopenia, nausea/vomiting, neurotoxicity, nephrotoxicity, diarrhea, and toxic death. QOL and grade 3 or 4 toxic profiles were secondary outcomes in the meta-analysis.

### Statistical analysis

The Cochrane Collaboration's Review Manager Software (RevMan Version 5.2, The Cochrane Collaboration, Copenhagen, 2014) was used to perform the meta-analyses. The overall effect size of each regimen was calculated as a weighted average of the inverse variance and 95 % confidence intervals (95 % CI) for study-specific estimates. The statistical heterogeneity among the individual studies was evaluated based on Cochrane's Q test and the I^2^ index, which express, as a percentage, the proportion of variability of the results due to heterogeneity as opposed to sampling error [26]. The presence of considerable heterogeneity was confirmed if I^2^ was > 75 % and if P < 0.10 [27]. A variance-based fixed effect model was applied to calculate the pooled effect. Where considerable heterogeneity was reported, the summary effects of the regimens were pooled using a random-effects model [28]. If appropriate, the heterogeneity was identified and explained using a subgroup analysis or sensitivity analysis [27]. A P-value less than 0.05 was considered significant for all statistical tests.

## RESULTS

### Search results

Table 1 contains a flowchart that describes the process by which we screened and selected trials. A total of 1320 relevant reports were initially retrieved from the electronic databases. After the removal of duplicates and after the titles and abstracts were screened, 74 publications met the inclusion criteria, and the full text for all 74 articles was available. Among these articles, 21 articles were excluded because they contained studies that were not RCTs; 12 articles were excluded because the included patients were not treated with a single chemotherapeutic agent, and intervention was preoperative or postoperative adjuvant chemotherapy combined with surgery; 25 articles were excluded because of inappropriate treatment comparisons; 2 articles were excluded because they were study protocols; and 1 article was excluded because it contained the same patient population as another study; 4 articles were excluded because of insufficient information for the current meta-analysis. In addition, a manual search of relevant references did not identify additional studies. Consequently, 9 trials that included patients with advanced NSCLC were ultimately eligible for inclusion in this meta-analysis.

**Table 1:**
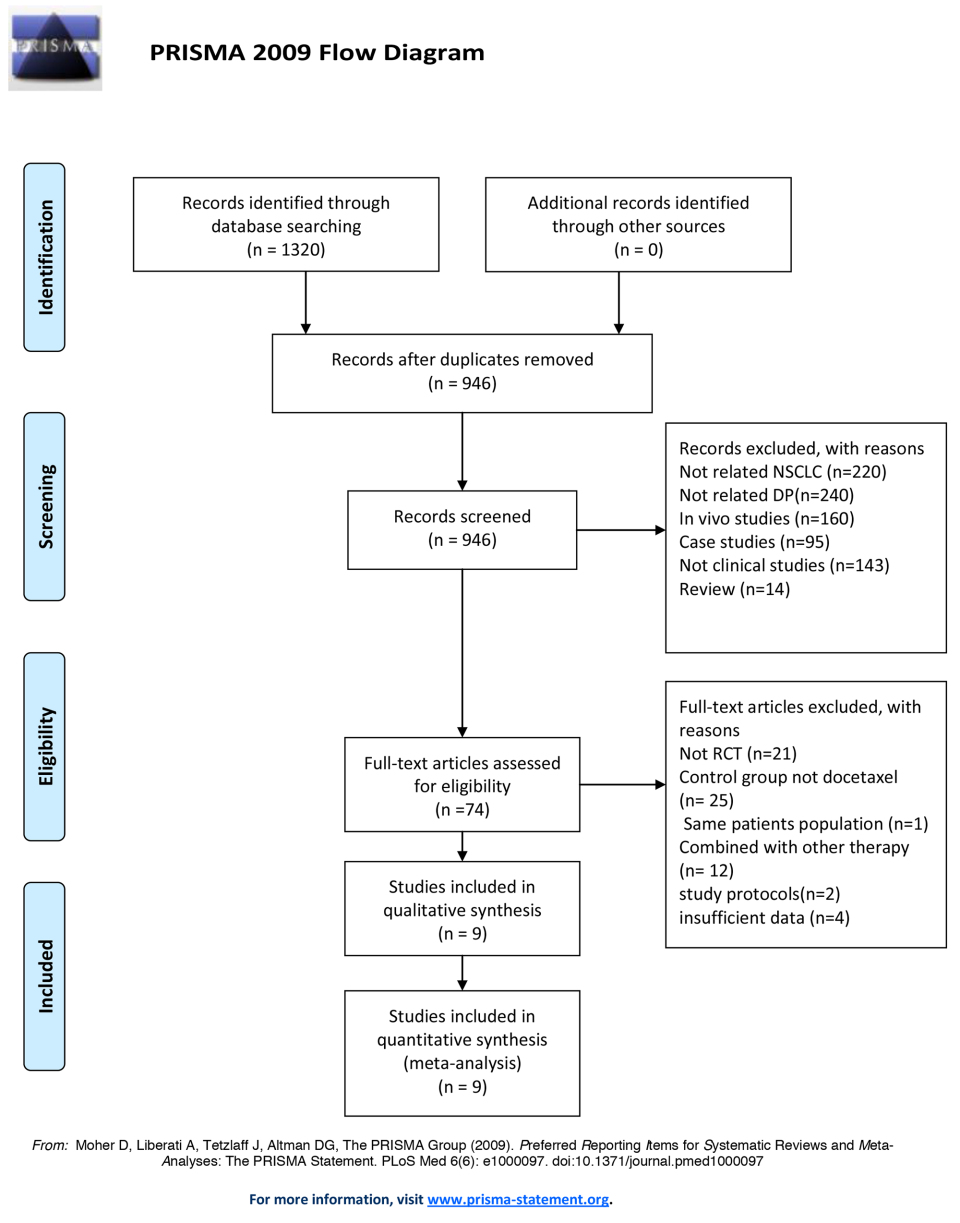
Flow diagram of the studies

### Characteristics of the trials

Detailed baseline characteristics of the patients included in the nine trials are listed in Table 2. In all, 1257 patients were randomized to receive the docetaxel monotherapy regimen (623 patients) or the DP combination doublet regimen (634 patients). Two trials [29, 30] were performed in Japan, one trial [16] was performed in Greece, and six trials [31–36] were performed in China. Two trials [16, 30] were randomized phase III trials. Five studies reported the permission of the ethics and scientific committees of the participating centers [16, 29–31, 33]. Two trials were registered in the Japan Clinical Oncology Group (JCOG, the former name of the West Japan Oncology Group [WJOG]) Data Center [29, 30]. One trial was conducted by the Lung Cancer Working Group of the Hellenic Oncology Research Group and was a prospective and multicenter trial [16]. None were placebo-controlled, double-blind trials. The patients in five trials [16, 29–31, 33] provided written informed consent before they underwent any study-related procedure. Survival data were well reported and were available in four studies [16, 29, 30, 34]. Response to therapy was reported in all nine studies. QOL assessment and symptom scores were evaluated in three trials [16, 29, 30]. The appropriate sample capacity was calculated before the trials were conducted in two studies [29, 30]. G-CSF support for the DP combination group was given in one trial [16]. Standard antiemetic treatment together with standard pre- and post-medication with dexamethasone or a 5-HT3 antagonist was given to patients in both groups in seven trials in cases of allergic reactions and docetaxel-associated fluid retention syndrome [16, 29, 31, 33–36]. The patients in the studies were middle-aged and elderly. The number of treatment cycles ranged from 1 to 18 in the docetaxel arm and from 1 to 9 in the DP arm. Patient baseline assessments, which consist of complete medical history and physical examination, were performed before the initiation of therapy, and measurable lesions were monitored throughout the trials [16, 29, 30, 33]. Three trials reported major reasons for dose reductions, treatment discontinuation or treatment termination [16, 29, 30]. Additional data were collected on the epidermal growth factor receptor (EGFR) mutation status (exon 19 deletion or L858R point mutation) and post-study treatments; an ad hoc analysis was performed in one study [30].

**Table 2:** Summary of characteristics in studies included

Study(year)	country	Demographics					Intervention	
		Number	Age(years)	Male/Female	ECOG PS	stage	D group	DP group
Tsukada (2015)	Japan	D: 63DP:63	D: 76(70-88)DP: 76(70-86)	D:49/14DP:48/15	0-1	IIIA/IIIB,IV	One cycle: D 25 mg/m^2^ infused over 60 min on days 1, 8 and 15; repeated every 4 weeks	One cycle: D 20 mg/m^2^ infused over 60 min plus P 25 mg/m^2^ infused over 15–20 min on days 1, 8 and 15. repeated every 4 weeks
Georgoulias (2004)	Greece	D:152DP:167	D: 63(41-77)DP: 63(33-76)	D:137/15DP:157/10	0-2	IIIB, IV	One cycle: D 100 mg/m^2^ in a 1-hour intravenous infusion; repeated every 3 weeks	One cycle: D 100 mg/m^2^ over a 1-hour intravenous infusion on day 1 and P 80 mg/m^2^ on day 2, G-CSF 150 γg/m^2^ subcutaneously from days 3 to 9
Abe (2015)	Japan	D:137DP:139	D: 76(70-87)DP: 76(76-86)	D:95/42DP:101/38	0-1	III, IV	One cycle: D 60 mg/m^2^ infused over 60 minutes on day 1;repeated every 3 weeks	One cycle: D 20mg/m^2^ infused over 60 minutes plus P 25mg/m^2^ infused over 15 to 20 minutes on days 1, 8, and 15 every 4 weeks
Guo (2015)	China	D: 45DP:45	D: 64.9(63-75)DP: 65.7(60-77)	D:28/17DP:30/15	Nr	Nr	One cycle: D 35 mg/m^2^ infused over 60 min on days 1, 8 and 15; repeated every 4 weeks	One cycle: D 35 mg/m^2^ infused over 60 min on days 1, 8 and 15 plus P 40mg infused over 60 min, on days 1, 2 and 3; repeated every 4 weeks
Liu (2010)	China	D: 40DP:32	Nr	Nr	Nr	III, IV,	One cycle: D 70 mg/m^2^ infused over 60 min on days 1, 8; repeated every 3 weeks	One cycle: D 35 mg/m^2^ on days 1, 8 plus P 25 mg/m^2^, on days 1, 2 and 3; repeated every 3 weeks
Zeng* (2013)	China	D: 42DP:43	D: 54.8±5.4DP: 56.2 ±6.6	D:22/20DP:22 /21	Nr	IIb, III, IV	One cycle: D 75 mg/m^2^ on day 1; repeated every 3 weeks	One cycle: D 75 mg/m^2^ on day 1 plus P 75 mg/m^2^, on day 1; repeated every 3 weeks
Zhang* (2015)	China	D: 68DP:68	D: 63.6±10.5 DP: 62.1 ±11.2	D:35 /33DP:38 /30	Nr	III, IV,	One cycle: D 75 mg/m^2^ on days 1, 8; repeated every 4 weeks	One cycle: D 75 mg/m^2^ on day 1 plus P 20mg, on days from 1 to 5; repeated every 4 weeks
Jing (2014)	China	D: 36DP:36	Nr	D:24 /12DP:26 /10	Nr	IIb, III, IV	One cycle: D 75-85 mg/m^2^ infused over 60 min on day 1; repeated every 3 weeks	One cycle: D 75 mg/m^2^ on day 1 plus P 25 mg/m^2^, on days from 1 to 3; repeated every 3 weeks
Wang (2011)	China	D: 40DP:41	D: 58 (37-70)DP: 60(40-70)	D:31 /9DP:30 /11	0-2	IIIB, IV	One cycle: D 75 mg/m^2^ on day 1; repeated every 3weeks	One cycle: D 75 mg/m^2^ on day 1 plus P 80 mg/m^2^ divided into 3 days; repeated every 3 weeks

Abbreviations: D, docetaxel; DP, docetaxel/cisplatin; G-CSF, granulocyte colony-stimulating factor; ECOG PS, Eastern Cooperative Oncology Group performance status. Age was presented as mdian (range).

*Age was presented as mean ± SD.

### Risk of bias assessment

According to the Cochrane Collaboration recommendation, randomization methods were reported in four trials [16, 30–32]. Randomization to each arm was accomplished by stratification according to age, PS, and stage of the disease [16]; institution, disease stage (III v IV or recurrence), and age [30]. The baseline characteristics of the patients were generally well balanced between the treatment arms in each trial. Otherwise, allocation concealments and comprehensive methodological processes, as well as the blinding of participants and personnel, were not reported. Sufficient details of withdrawals and dropouts were described in all 4 studies. Two studies used the intention-to-treat approach in the handling of data [16, 30]. In the majority of the studies, whether enrollment of the participants was actually consecutive or not was unclear, and a selection bias could be completely excluded. The details of the risk of bias are illustrated in Supplementary Figure 1.

### Response

**Figure 1:**
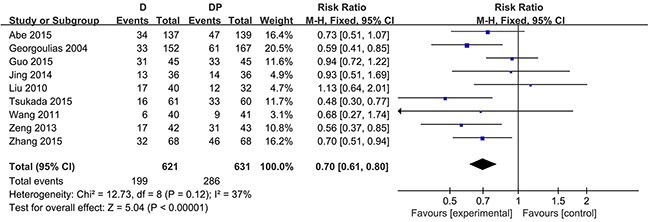
Forest plot for overall response

Data on the objective response rates were available in all nine trials. The meta-analysis demonstrated that the combination chemotherapy regimens of DP had a higher overall response rate (CR plus PR) compared with the docetaxel regimen (RR = 0.70; 95 % CI, 0.61 to 0.80; P < 0.00001). Additionally, no considerable heterogeneity was found (χ^2^ = 12.73, I^2^ = 37 %, P = 0.12) (Figure 1). In the case of G-CSF, a sensitivity analysis revealed that the overall response rate was also in favor of the combination chemotherapy regimen of DP (RR = 0.73; 95 % CI, 0.63 to 0.84; P < 0.0001; χ^2^ = 11.13, I^2^ = 37 %, P = 0.13). CR and PR were reported in detail in eight trials [16, 29, 31–36]. In terms of PR, the results were consistent with the overall response rate, as the RR estimates for eight trials favored the combination chemotherapy regimens (RR = 0.71, 95 % CI = 0.59 to 0.86, P = 0.0004; heterogeneity: χ^2^ = 7.16, P = 0.41, I^2^ = 2 %) (Supplementary Figure 2). Moreover, the DP regimen yielded superior CR rates compared with docetaxel monotherapy (RR = 0.64, 95 % CI = 0.45 to 0.92, P = 0.01; heterogeneity: χ^2^ = 4.15, P = 0.53, I^2^ = 0 %) (Supplementary Figure 3). The overall number of patients who achieved a CR was less than the number who achieved a PR. Particularly, no patients achieved a CR in either of the two arms in two trials [33, 35].

### Survival

**Figure 2:**
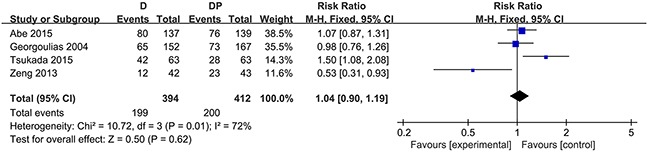
Forest plot for one-year survival rate

The one-year survival rate was stated in four trials [16, 29, 30, 34]. No statistically significant difference was observed between the docetaxel monotherapy group and the DP group in terms of the one-year survival rate (RR = 1.04; 95 % CI, 0.90 to 1.19; P = 0.62). Substantial heterogeneity was found among the trial estimates (χ^2^ = 10.72, P = 0.01), and the I^2^ index indicated that 72 % of the variability across trials was due to heterogeneity rather than chance (Figure 2). Three trials [16, 29, 30] revealed no significant difference between the two groups with regard to the 1-year survival rate, yet one trial [29] showed that the OS was considerably worse in the docetaxel group (hazard ratio for DP over docetaxel, 0.23; 95 % CI, 0.09 to 0.62) for patients 70 – 74 years of age. One trial reported a higher 1-year survival rate in the DP group. In the case of G-CSF, age (≦75), and performance status (≦ 2) in one trial conducted by Georgoulias, the sensitivity analysis revealed that patients in the DP group did not seem to experience an increased survival at 1 year (RR = 1.01, 95 % CI, 0.65 to 1.56, P = 0.98; χ^2^ = 10.18, P = 0.006, I^2^ = 80 %).

### Toxicity

Toxicity profile results with respect to the frequency of NCI-CTC grade 3 – 4 side effects were available for all trials. The number of trials with data available for anemia, thrombocytopenia, nausea/vomiting, nephrotoxicity, hyponatremia and treatment-related deaths was 7, 7, 6, 3, 2 and 4, respectively. The reporting of side effects was heterogeneous among trials. Patients who were treated with the DP regimen were more likely to experience anemia (RR = 0.34, 95 % CI = 0.19 to 0.61, P = 0.0002), thrombocytopenia (RR = 0.27, 95 % CI = 0.12 to 0.59, P = 0.001), and nausea/vomiting (RR = 0.43, 95 % CI = 0.24 to 0.75, P < 0.003) compared with patients who were treated with docetaxel. Moreover, the DP regimen led to more frequent grade 3 or 4 nephrotoxicity (RR = 0.06, 95 % CI = 0.01 to 0.45, P = 0.006), hyponatremia (RR = 0.47, 95 % CI = 0.25 to 0.88, P = 0.02), and treatment-related deaths (RR = 0.19, 95 % CI = 0.04 to 0.86, P = 0.03), and this difference was statistically significant. Almost all treatment-related deaths occurred in the DP treatment group with the exception of one patient who died of febrile neutropenia in the docetaxel group in one trial [16]. In addition, two trials [29, 30] demonstrated that the primary cause of treatment-related deaths was pneumonitis, while the trial [16] performed by Georgoulias showed that the causes of causes were diverse and were primarily febrile neutropenia, acute renal failure, febrile diarrhea and vomiting, grade 3 thrombocytopenia, pulmonary infection, anemia and non-neutropenic infection.

**Figure 3:**
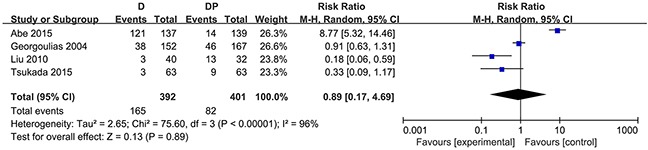
Forest plot for neutropenia

**Figure 4:**
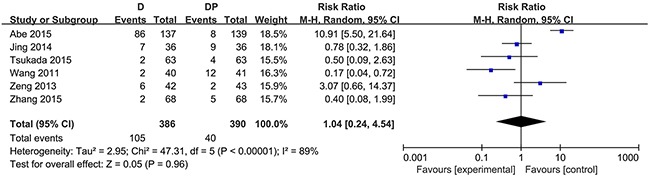
Forest plot for leucopenia

The risk of grade 3 or 4 neurotoxicity was comparable between the two modalities (RR = 0.33, 95 % CI = 0.09 to 1.18, P = 0.09). Four studies assessed the occurrence of neutropenia. Considerable heterogeneity existed in the morbidity associated with neutropenia among the 4 studies (χ^2^ = 75.60, P < 0.00001, I^2^ = 96 %), and the random effects model was applied to perform the data analysis. Otherwise, no statistically significant difference in the morbidity associated with neutropenia was observed (RR =0.89, 95 % CI = 0.17 to 4.69, P = 0.89) (Figure 3). Considering the role of G-CSF, a sensitivity analysis was performed and found no apparent significant difference in the incidence of grade 3 or 4 neutropenia between the two arms (RR = 0.85, 95% CI = 0.05 to 13.65, P = 0.91; χ^2^ = 50.81, P < 0.00001, I^2^ = 96 %). The results were also consistent in the morbidity associated with febrile neutropenia (RR = 1.68, 95 % CI 0.08 to 35.47, p = 0.74; χ^2^ = 12.12, P = 0.002, I^2^ = 83 %). Data on leucopenia were available in six trials. Similarly, pooled data showed that no statistically significant difference existed with respect to leucopenia (RR = 1.04, 95% CI = 0.24 to 4.54, P = 0.96; χ^2^ = 47.31, P < 0.00001, I^2^ = 89 %) (Figure 4).

Diarrhea, fatigue and pneumonitis were evaluated separately in 5, 3, and 2 trials, respectively. Overall, the meta-analysis did not reveal that patients in the DP chemotherapy group experienced a greater incidence rate of the above-mentioned side effects compared with the docetaxel group. Mucositis was reported in two trials [16, 36]. Pooled data revealed a higher incidence rate of mucositis in patients who received the DP regimen (RR = 0.39, 95 % CI 0.22 to 0.70, P = 0.001; χ^2^ = 1.90, P = 0.17, I^2^ = 47 %). A summary of hematological and non-hematological events is presented in Table 3.

**Table 3:** Summary of toxicity meta-analyses comparing D and DP regimens

Analyses	No. of trials	P-value for homogeneity	Model	RR (95%CI)	P-value
Anemia	7	0.10	F	0.34(0.19-0.61)	0.0002
Neutropenia	4	<0.00001	R	0.89(0.17 -4.69)	0.89
Febrile neutropenia	3	0.002	R	1.68 (0.08-35.47)	0.74
Thrombocytopenia	7	0.25	F	0.27(0.12-0.59)	0.001
Nausea/vomiting	6	0.02	F	0.43(0.24-0.75)	0.003
Neurotoxicity	2	-	F	0.33(0.09-1.18)	0.09
Nephrotoxicity	3	0.52	F	0.06 (0.01-0.45)	0.006
Treatment-related deaths	4	0.88	F	0.19(0.04-0.86)	0.03
Diarrhea	5	0.05	F	0.60(0.32-1.13)	0.11
Hyponatremia	2	0.24	F	0.47(0.25-0.88)	0.02
leukopenia	6	<0.00001	R	1.04 (0.24 -4.54)	0.96
Mucositis	2	0.17	F	0.39 (0.22-0.70)	0.001
Fatigue	3	0.31	F	0.68 (0.28-1.63)	0.38
Pneumonitis	2	0.06	F	3.43(0.85-13.88)	0.08

Abbreviations: RR, risk ratio; R, random effects model; F, fixed effects mode; CI, confidence interval.

### Quality of life

Three trials [16, 29, 30] included a QOL assessment. As the data were not available for quantitative synthesis, we incorporated a review here. Symptom score questionnaire responses were assessed after each treatment cycle until the end of the third cycle in one trial [16, 30] and were assessed at baseline, at the end of the third cycle, and at the end of chemotherapy (EOC) in one trial [16]. The compliance for QOL assessment was considered generally high. All three trials reported the numbers of patients with missing data for the QOL assessment. Two trials reported the reason for withdrawal from the QOL assessment during the treatment course. One trial [29] reported least square mean scores at baseline and at 8 weeks and found no significant difference between the groups (two-sided, P = 0.564, ANCOVA with the baseline score as a covariate). Additionally, one trial [16] reported no significant differences in QOL assessments at the end of the third cycle and at the EOC between the two arms. The trial conducted by Abe [30] reported that the mean total score remained near its baseline value in the docetaxel arm after the third cycle of therapy, while the mean total score gradually declined in the DP arm, and a significant difference was observed between the score at baseline and the score at the end of cycle 3 (P < 0.1).

## DISCUSSION

Overall, many clinical trials have confirmed the activity, efficacy and toxicity of the DP combination in patients with advanced NSCLC [37–39]. It was reported that combination chemotherapy led to a nearly two-fold increase in the response rate and a modest improvement in the one-year survival rate, with increased toxicity profiles compared with single-agent treatment in patients with NSCLC [40]. In fact, the results of this meta-analysis supported, to some extent, the above findings. The present meta-analysis showed that the DP combination chemotherapy regimens had a higher overall response rate (PR plus CR) than the docetaxel regimen despite the observation that most patients achieved a partial response. However, a survival advantage was not found in the DP combination arm after an analysis of the 1-year survival rate. It appears that toxic effects, including anemia, nausea/vomiting, thrombocytopenia, hyponatremia, mucositis, nephrotoxicity and treatment-related deaths, are more likely to result from DP doublet regimens. The occurrence of toxic effects was in accordance with that in previous studies. According to our analyses, no significant difference in the occurrence of other side effects was established between these two arms, as only a slight difference was noted [21, 40, 41].

Cisplatin-based doublet regimens were associated with a higher response rate (PR plus CR) and lower occurrence of stable disease compared with non-platinum-based doublet regimens [42]. On the contrary, this suggested that an increased survival at 1 year might be the result of a better response to cisplatin. In advanced NSCLC, an improvement in survival without severe treatment-related toxicity is a major challenge in the management of patients with this cancer type. The results of this meta-analysis were consistent with those of the trial conducted by Georgoulias in 2004 [16]. A higher response rate was achieved in patients who received the DC regimen but without improvement in the one-year survival rate. Grade 3/4 nausea/vomiting (P = 0.0001), nephroroxicity (P = 0.006), neurotoxicity (P = 0.017) and diarrhea (P = 0.007) were significantly more common in the DC group. As an alternative, docetaxel could be a reasonable option for patients who cannot tolerate cisplatin in terms of QOL, especially for elderly patients with NSCLC with poor PS. Nevertheless, even though cisplatin-based doublet regimens resulted in a higher incidence rate of toxic effects, the comprehensive appraisal seems to still favor cisplatin-based regimens. The QOL profile comprises the overall performance of activity, efficacy and toxicity of chemotherapy regimens, and in turn, may be associated with the mode of treatment delivery. A higher incidence of side effects was the main cause of the decline in the QOL score. The declining QOL score was responsible for treatment discontinuation and a low progression-free survival (PFS). Otherwise, the lack of significant difference in the QOL between the two groups in three trials seemingly conflicted with the greater toxicity profiles in the DP arms. Belani performed an analysis on an elderly subgroup in the TAX 326 trial, and found that similar activity was achieved with first-line DP chemotherapy in younger and elderly patients with advanced NSCLC. Although slightly more toxicity occurred in older patients compared with younger patients, elderly patients tolerated the DP regime well [43]. However, elderly patients were sufficiently represented in the above clinical trial, which means that it may be unreasonable to extrapolate that result to the general elderly population.

The DP regimen has been studied as an initial therapy for advanced, metastatic and recurrent NSCLC. The acknowledged and recommended total dose/cycle for the two drugs is 75 mg/m^2^ administered on a single day. Certainly, many variations exist in dose management [19]. Mitsudomi reported that the DP regimen was administered as docetaxel 60 mg/m^2^ plus cisplatin 80 mg/m^2^ on day 1 every 21 days in an open label, randomized phase 3 trial that compared gefitinib versus DP for NSCLC [44]. It was reported that when docetaxel was given weekly rather than once every 3 weeks, the risk of neutropenia was reduced, while the antitumor activity appeared to be maintained [45]. In the present meta-analysis, the DP regimens of nine trials were diverse. DP regimens were administered as D 60 mg/m^2^ plus P 75 mg/m^2^ in one cycle in two trials [29, 30] and as D 100 mg/m^2^ plus P 80 mg/m^2^ in one trial [16]. Out of six Chinese studies, both drugs were administered as 75 mg/m^2^ on a single day in only one study [34]. Dosing schedules, especially dosing intervals and dosing sequences, might have different effects on toxicity and antitumor effects. Myelosuppression and nephrotoxicity caused by cisplatin were dose-limiting factors, and severe nephrotoxicity was potentially fatal [46]. The reduction of adverse effects and the improvement of antitumor effects as much as possible formed the basic principle of the trials. Using a C57BL/6N Lewis lung carcinoma mouse model, Kodama [46] found that a sequential D-P regimen in which cisplatin was administered 12 h after docetaxel, not only inhibited tumor growth to a great extent but also significantly reduced treatment-related deaths and renal toxicity compared with the DP regimen that was simultaneously used. This corresponded to the results of an in-vitro study [47]. It was reported that the enhanced cytotoxicity of a sequential D-P regimen was attributed to the accumulation of intracellular Pt–glutathione complexes, as docetaxel appeared to suppress the up-regulation of multidrug resistance-associated protein-1 (MRP-1) induced by cisplatin exposure [48]. Otherwise, docetaxel is active in patients who are refractory or resistant to cisplatin, and produces responses that range from 18 % to 25 %; this implies the lack of crossover in the mechanisms of action between docetaxel and cisplatin [49].

Dosage reductions or therapy adjustments need to be implemented after the occurrence of grade 3 or 4 toxicities, but not for grade 1 or 2 toxicities. Therefore, the data on grades 3 or 4 toxicities were quantitatively synthesized in the meta-analysis. Hematologic adverse events were the most common major toxicities because of myelosuppression. Accepted practice guidelines suggested that prophylactic use of colony stimulating factors (CSFs) could lead to a greater than 20 % reduction in the incidence of febrile neutropenia when given with antineoplastic regimens [50]. The use of CSFs was considered for regimens with an incidence of febrile neutropenia between 10 % and 20 %, but was not recommended when the incidence was less than 10 % [50]. With regard to grade 3/4 neutropenia or febrile neutropenia, no significant difference was found between the two groups, which might be attributed to the prophylactic use of CSF in the DP arm in that trial [16].

Efficacy may not be the only factor that might affect a physician's decision with regard to the choice of chemotherapy regimen for NSCLC. The principal goals of treatment should be palliation, an acceptable quality of life and prolonged survival. Consequently, clinicians should carefully define the best anticancer drug, schedule of administration, and treatment strategy, depending on potential toxicity and the individual patient's wishes. Some new therapies have been explored in recent years. It has been suggested that matrix metalloproteinase (MMP) inhibitors could significantly reduce vascular density around lung tumors and that the tumor growth inhibition rate could reach 88 % [36]. Thalidomide has anti-angiogenesis effects and has insignificant toxicity. Preliminary data have confirmed the feasibility of thalidomide use for advanced NSCLC. Docetaxel is not soluble in water, which reduces local dosage and its clinical effect. Currently, research that focuses on new formulations of docetaxel has become a hot topic. The new formulations can improve water solubility, reduce adverse reactions, and improve the utilization rate. Researchers have found that docetaxel packed with liposomes in an oil phase or a microemulsion system might result in satisfactory clinical effects [31].

The limitations of this systematic review involve the uniformity of the administration program and the small size of the included RCTs. Given the special features of lung cancer, imbalances in baseline prognostic factors and post-protocol treatment, such as epidermal growth factor receptor (EGFR) mutations, pathological type and comorbid illnesses, may be responsible for unexpectedly large differences in some measures between treatment arms. More patients with non-squamous histology were included in the DP arm than in the docetaxel arm in the trial conducted by Tsukada [29]. Active EGFR mutations are often observed in female patients or in patients with adenocarcinoma and have been reported as a favorable prognostic factor in patients with NSCLC [51, 52]. It was reported in two phase III studies that patients with EGFR gene mutations had increased survival and response rates when treated with docetaxel or gefitinib, compared with patients with wild-type EGFR [53, 54]. Additionally, we used the Cochrane Collaboration's tool to assess the risk of bias in order to evaluate the methodological quality of the included trials; we also conducted a sensitivity analysis accordingly. Trials with a high or unclear risk of bias could lower the quality of evidence in our results [55]. The reports of the toxicity profiles were very heterogeneous, the causes of which are diverse. Consequently, caution should be taken when estimates of the meta-analysis are interpreted.

## CONCLUSION

The DP regimens led to a higher overall response rate in comparison with docetaxel regimens. In addition, unlike the promising survival and favorable toxicity profile seen in many other studies with the DP regimen [56, 57], a survival advantage was not observed with the DP regimen. Moreover, DP doublet regimens seemed to be associated with higher toxicity, including anemia, nausea/vomiting, thrombocytopenia, hyponatremia, mucositis and nephrotoxicity and led to more treatment-related deaths. Although the interpretation of the study results was limited, we believe that to a certain extent, our analyses may provide valuable information for physicians who need to decide the best treatment strategy among all the possible regimens for patients with NSCLC. However, more powered studies with much larger sample sizes are advocated in order to obtain a more concrete conclusion.

## SUPPLEMENTARY MATERIALS FIGURES



## Abbreviations

NSCLC: Non-small-cell lung cancer
DP: docetaxel plus cisplatin
CNKI: China National Knowledge Internet
QOL: quality of life
OS: overall survival
PR: partial response
CR: complete response
EGFR: epidermal growth factor receptor
MMP: matrix metalloproteinase
PFS: progression-free survival
CSFs: colony stimulating factors
MRP-1: multidrug resistance-associated protein-1
RCTs: randomized clinical trials
