# Supplementary material for: Docetaxel versus docetaxel plus cisplatin for non-small-cell lung cancer: a meta-analysis of randomized clinical trials
Source: Oncotarget. 2017 Apr 13;8(34):57365–78. doi: 10.18632/oncotarget.17071 (PMC5593648; doi:10.18632/oncotarget.17071)
Supplement: Supplementary file 1 [file oncotarget-08-57365-s001.pdf]

Docetaxel versus docetaxel plus cisplatin for non–small-cell lung cancer: a meta-analysis of randomized clinical trials

SUPPLEMENTARY MATERIALS

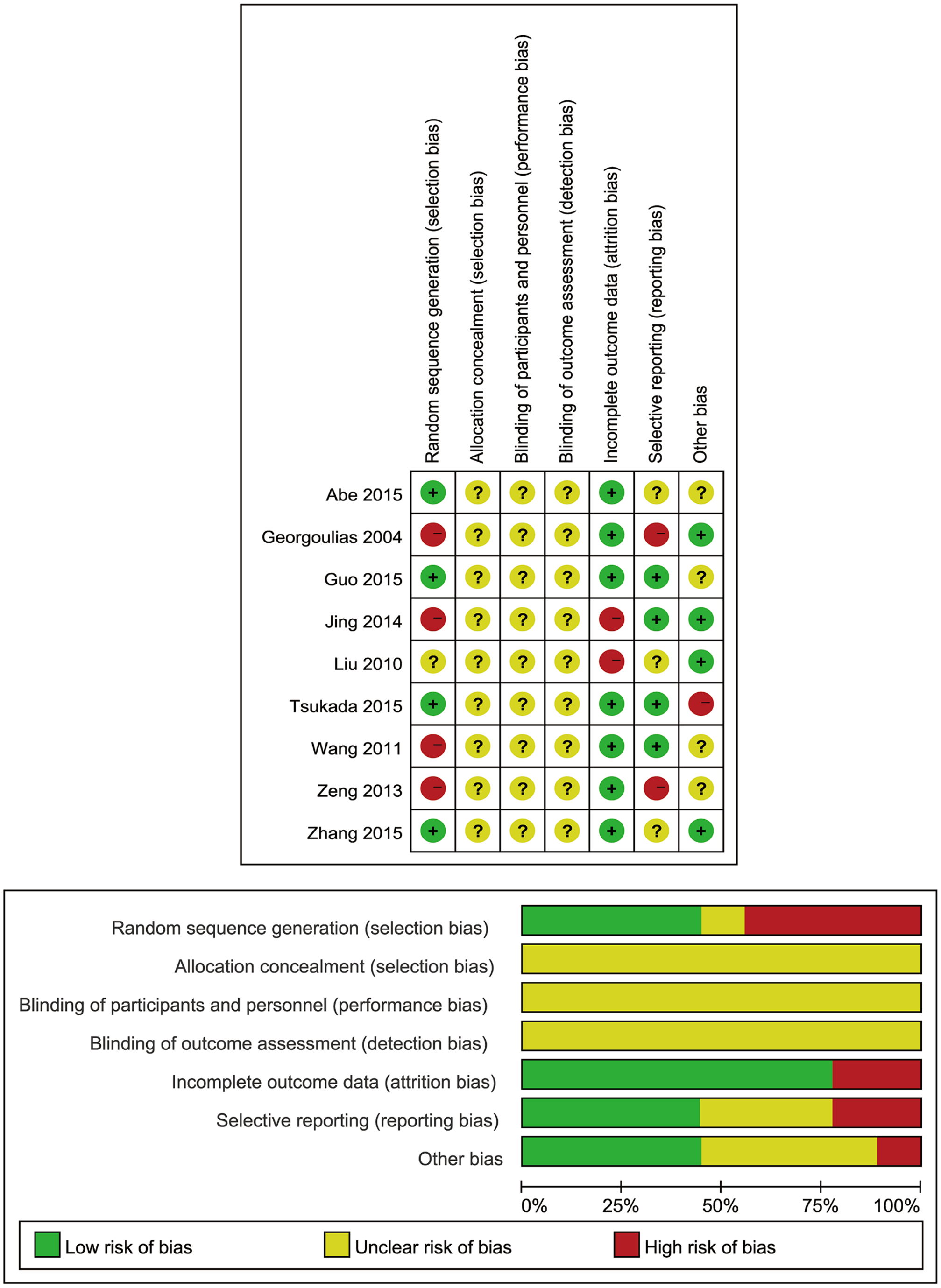

Supplementary Figure 1: Risk of bias summary and graph.

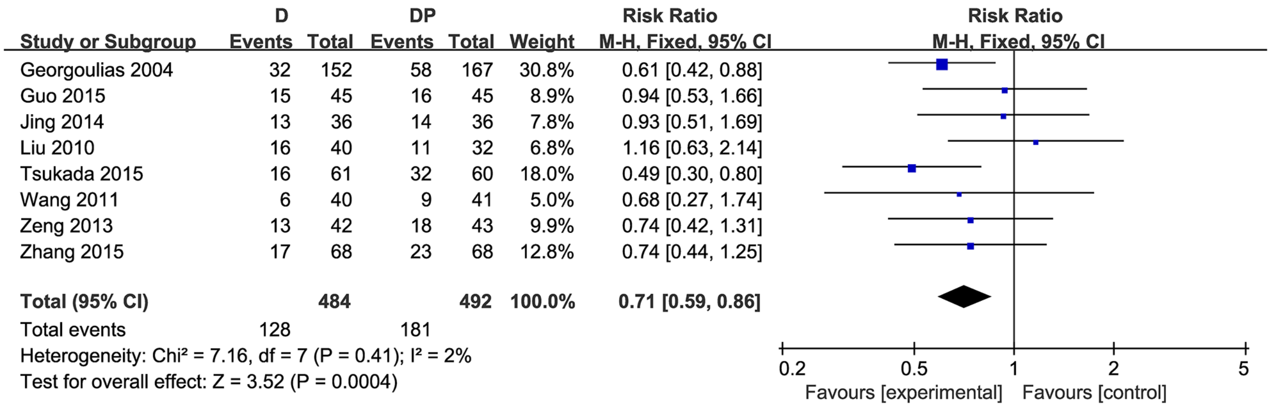

Supplementary Figure 2: Forest plot for partial response.

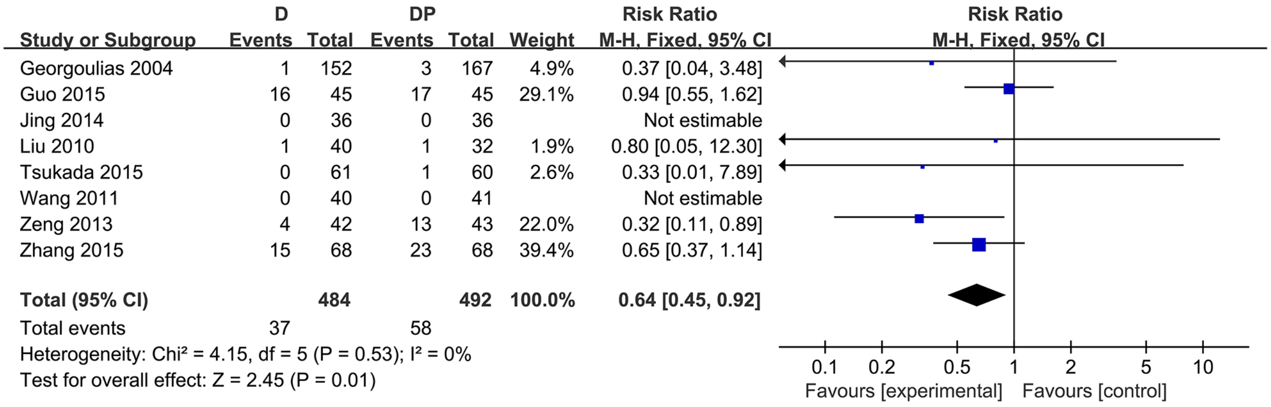

Supplementary Figure 3: Forest plot for complete response.
